# Attitudes of healthcare providers and patients/families toward a family-centered visitation and care program: a qualitative study

**DOI:** 10.3389/fmed.2025.1655210

**Published:** 2026-04-02

**Authors:** Ke Chen, Caiyun Zhang, Yuchen Wu, Weigang Yue, Xiubin Zhang, Lulu Han, Zhigang Zhang

**Affiliations:** 1 Sichuan Provincial Center for Mental Health, Sichuan Provincial People’s Hospital, School of Medicine, University of Electronic Science and Technology of China, Chengdu, China; 2 School of Nursing, Lanzhou University, Lanzhou, China; 3 First Hospital of Lanzhou University, Lanzhou, China

**Keywords:** cognition, family-centered, Intensive Care Unit, qualitative research, visitation

## Abstract

**Design:**

Patients admitted to the Intensive Care Unit (ICU) often experience severe health complications, which results in restricted ward access and limited family visitation opportunities. This frequently leaves families inadequately informed about the patient’s condition. The Patient and Family-Centered Visitation (PFCV) model promotes a more open and flexible visitation approach, emphasizing patient autonomy and fostering an environment of mutual respect and trust among healthcare providers, patients, and families. Currently, no hospitals in China have implemented a fully family-centered visitation system.

**Aim:**

To understand the evolving landscape of family-centered visitation in China, this study aimed to explore the advantages, disadvantages, and implementation potential of such a system in clinical practice from the perspectives of healthcare professionals (doctors and nurses), patients, and their families.

**Results:**

Using a phenomenological qualitative approach, this study involved physicians, nurses, patients, and family members from a tertiary hospital in Lanzhou City. Data were organized and coded using the qualitative analysis software NVivo 11.0 Plus and were subsequently analyzed through Colaizzi’s seven-step method to derive final themes.

**Conclusion:**

The establishment of a family-centered visitation system in the ICU faces significant challenges related to workforce, environmental constraints, and management. However, opportunities exist for family involvement in certain aspects of patient care. To address these challenges effectively, hospital administration must provide tailored support for implementing a context-appropriate family-centered visitation system.

## 1 Background

The discipline of critical care medicine merged and gained international recognition in the late 1970s. During this period, China also began establishing Intensive Care Units (ICUs) to manage critically ill and medically complex patients (1). Despite the undeniable role of ICUs in preserving life, several persistent challenges continue to compromise long-term patient outcomes. During and after ICU hospitalization, patients and their families may develop symptoms of Intensive Care Unit Syndrome (ICUS) and Post-Intensive Care Syndrome (PICS) (2), conditions characterized by new or worsening impairments in cognitive, psychological, and physical domains (3). Parallel to the broader shift from a conventional biomedical model toward a bio-psycho-social approach, critical care medicine has increasingly focused on developing novel ICU management strategies aimed at improving recovery trajectories. Within this evolving landscape, significant emphasis is now placed on reforming visitation policies and promoting active family involvement in the care process (1). Consequently, the healthcare delivery model is progressively shifting toward a tripartite collaborative partnership among clinicians, patients, and families, with the overarching goal of enhancing health outcomes and improving the quality of care (3). Such collaboration is particularly essential for patients with impaired self-care abilities (4).

Patient- and Family-Centered Care (PFCC) in the ICU is a healthcare planning, delivery, and evaluation approach founded on mutually beneficial partnerships among healthcare providers, patients, and families (5). It emphasizes that both patients and their families are vital partners in ensuring quality and safety, and that patients’ healthcare decisions should be integrated with their broader life experiences. Implementing PFCC-based visitation in the ICU has been demonstrated to effectively improve health outcomes and care experiences for patients and their families, while also increasing overall satisfaction. This model is particularly well-established in specialties such as neonatology, pediatrics, and oncology (6–8).

PFCC-based visitation has received formal endorsement from international organizations such as the Institute of Medicine (9), the Society of Critical Care Medicine (10), and the American Academy of Pediatrics (11), and has been widely adopted and promoted across North America and Europe. However, its reporting and implementation in China have remained limited due to factors such as differences in healthcare environments and clinician-patient relationships, constraints in human resources, and insufficient understanding of its underlying principles (12–14). In Chinese ICUs, clinical attention remains largely focused on patients’ physical symptoms, with limited consideration given to the psychological and social needs of both patients and their families. Furthermore, healthcare professionals rarely receive formal education on PFCC, resulting in an incomplete grasp of its core principles. In this context, this study employs a qualitative research design, using in-depth interviews to explore the perspectives of healthcare providers, patients, and family members regarding family-centered visitation, with the aim of informing the development of a visitation model suited to the Chinese setting.

Based on the above considerations, this qualitative study aims to explore the perceptions and experiences of key stakeholders regarding the implementation of a family-centered visitation policy in the ICU within the Chinese context. To achieve this aim, the following research questions were formulated:

1 What are the overall perceptions—including perceived benefits and concerns—of physicians, nurses, patients, and family members regarding a family-centered visitation policy in the ICU?
2 What key prerequisites and potential barriers—related to environment, management, and resources—are perceived as necessary for the successful implementation of such a policy?
3 How do stakeholders envision the roles of family members in patient care, communication, and decision-making within a family-centered visitation model?

## 2 Methods

This study adopted a qualitative research design underpinned by a descriptive phenomenological approach. Descriptive phenomenology emphasizes “returning to the things themselves,” focusing on participants’ lived experiences in order to gain an in-depth understanding of their subjective experiences. The reporting of this study was conducted in accordance with the Consolidated Criteria for Reporting Qualitative Research (COREQ) checklist.

### 2.1 Overview and setting

This study employs a qualitative phenomenological approach to explore the perceptions of healthcare professionals and patients in the Intensive Care Unit (ICU) toward the family-centered visitation system. The perspectives of physicians, nurses, patients, and their families are shaped by their distinct cultural backgrounds, traditions, family environments, and socioeconomic circumstances. It is therefore essential for participants to articulate their own views and lived experiences, necessitating that the researcher engage deeply with them to understand these firsthand accounts.

A purposive sampling technique was used to recruit doctors, nurses, patients, and family members from a tertiary care hospital in Lanzhou City between January and June 2024. Following approval from the Ethics Committee of [REDACTED], the researcher communicated the study objectives and significance to potential participants. Individuals who voluntarily agreed to participate were selected, and their informed consent was obtained prior to data collection.

The sample size was determined in accordance with the principle of data saturation, a common criterion in qualitative research. Saturation refers to the point at which data collection ceases, when no new information or emergent themes are identified in subsequent interviews.

### 2.2 Inclusion and exclusion criteria

#### 2.2.1 Healthcare professionals

- Inclusion criteria comprised:

◦ Currently employed as a physician or registered nurse in an ICU.
◦ A minimum of two years (≥2 years) of clinical experience in intensive care medicine.

- Exclusion criteria included:

◦ Trainees (e.g., interns, residents) or advanced practice providers.
◦ Individuals who declined participation or withdrew consent.

#### 2.2.2 Patients

- Inclusion criteria were as follows:

◦ Age ≥18 years.
◦ ICU length of stay ≥72 h at the time of recruitment.
◦ Capacity to provide verbal consent and communicate effectively.

- Exclusion criteria comprised:

◦ Deemed clinically unstable by the treating team.
◦ Declined participation or withdrew consent.

#### 2.2.3 Family members

- Inclusion criteria required:

◦ Age ≥18 years.
◦ Ability to communicate effectively in Chinese.
◦ Prior visitation experience within the ICU (≥1 visit).

- Exclusion criteria were:

◦ Presence of severe cognitive impairment or a diagnosed psychiatric condition that could hinder cooperation.
◦ Refusal to participate or withdrawal of consent.

### 2.3 Data collection

A semi-structured interview guide (see Appendix) was utilized for data collection during in-person interviews. The guide was developed by the research team based on the study objectives and a comprehensive review of relevant domestic and international literature. The initial version was refined through consultations with experts in qualitative nursing research and was subsequently piloted with three individuals from each stakeholder group (physicians, nurses, patients, and family caregivers). The final guide was adjusted based on insights from this pilot phase.

All interviews were conducted by graduate students formally trained in qualitative research methods, assisted by research assistants tasked with documenting non-verbal cues (e.g., tone of voice, facial expressions, body language). Prior to each interview, written informed consent was obtained. Sessions were audio-recorded in their entirety using iFlytek recording software.

Each interview session commenced with a brief overview of the study background. The conversation then followed the semi-structured guide, allowing flexibility for probing and follow-up questions. To ensure privacy and an environment conducive to open discussion, all interviews were conducted in quiet, private consultation rooms. Each participant was interviewed once, with sessions lasting between 30 and 50 min (mean duration: 35 min).

### 2.4 Data analysis

Data analysis adhered to Colaizzi’s seven-step phenomenological method. All interview recordings were transcribed verbatim within 24 h, with contextual annotations added during transcription. The textual data were then imported into NVivo 14.0 software to facilitate systematic management and analysis. The analytical procedure included the following steps:

(1) Familiarization: Repeated listening to audio recordings, verbatim transcription, and immersive reading of transcripts.
(2) Identifying Significant Statements: Extracting statements pertinent to family-centered care in the ICU.
(3) Formulated Meanings: The underlying meanings of key phrases and statements were interpreted and coded.
(4) Clustering Themes: Related codes were grouped into sub-themes, and similar sub-themes were synthesized into overarching themes.
(5) Exhaustive Description: The findings were integrated into a comprehensive narrative that captures the essence of the phenomenon.
(6) Producing the Fundamental Structure: A descriptive model of family-centered care in the ICU was constructed and refined.
(7) Verification: The credibility of the findings was enhanced through member checking and peer review.
(8) Confidentiality: To ensure participant confidentiality, an anonymized coding system was employed. Patients, family members, physicians, and nurses were assigned the prefixes P, F, D, and N, respectively, each followed by a unique numerical identifier.

### 2.5 Ethical considerations

This study received ethical approval from the Ethics Committee of the Lanzhou University School of Nursing (Ethics Code: LZUHLXY20200015). Prior to each interview, written informed consent was obtained from all participants after a detailed explanation of the study’s purpose and procedures. Participants were explicitly informed of the voluntary nature of their involvement and their right to withdraw at any time without affecting their clinical care.

It was acknowledged that the primary interviewer’s dual role as an ICU nurse could introduce bias, due to her pre-existing professional relationships and the inherent power dynamics with fellow nurses, patients, and their families. The study incorporated specific strategies in its design to mitigate these risks. At the outset of each interview, the interviewer explicitly clarified her role as a “researcher” distinct from a “care provider,” ensured confidentiality, and emphasized that their responses would not influence their clinical treatment. Accordingly, the interviews were structured to focus solely on listening to and understanding participants’ personal experiences and perspectives, without any form of clinical assessment.

Furthermore, to ensure analytical rigor and minimize individual bias, the research team held regular collaborative discussions during the data analysis phase. These discussions aimed to critically examine emerging interpretations, ensuring that the findings were grounded in the data and not merely reflective of the interviewer’s pre-existing clinical assumptions.

## 3 Results

### 3.1 Participant characteristics

#### 3.1.1 Physicians and nurses

This study enrolled nine Intensive Care Unit (ICU) physicians and seventeen ICU nurses based on the predefined inclusion and exclusion criteria. The physicians (5 males, 4 females) were aged 33 to 47 years (mean = 39.4 years) and had an average of 12.9 years of ICU experience. Five physicians held doctoral degrees, three held master’s degrees, and one held a bachelor’s degree. The nurses (7 males, 10 females) were aged 28 to 40 years (mean = 32.4 years) and had an average of 9.4 years of ICU employment. In terms of educational background, five held master’s degrees, ten held bachelor’s degrees, and two held specialist qualifications.

#### 3.1.2 Patients

The study included ten ICU patients (8 males, 2 females). They were aged 21 to 75 years (mean = 44.4 years), with an average ICU length of stay of 5.5 days.

#### 3.1.3 Family members

The study also involved sixteen family members of ICU patients (12 males, 4 females). Their ages ranged from 20 to 60 years (mean = 38.3 years). On average, they spent 6.3 days in the ICU and approximately 20.25 h per day accompanying their relatives outside the ward.

### 3.2 Result analysis

Thematic analysis was conducted, informed by the core principles of patient- and family-centered care, to analyze the data and synthesize themes. This process culminated in the identification of three primary themes and eleven corresponding sub-themes, which are presented in Table 1.

**Table 1:** Theme and subtheme.

Theme	Subtheme
Respect	The needs of patients and their families
	Environmental facilities
	Social support
Communication and information sharing	ICU diary
	Family coordinator
	Family meetings
Participation	Daily care
	Participate in early rehabilitation exercises
	Participate in decision-making
	Clinical rounds

### 3.3 Theme 1: respect

All participants emphasized the need to uphold the fundamental rights of patients and their families, protect patient privacy, and gain a thorough understanding of the patient’s family dynamics, needs, values, beliefs, and cultural background before implementing family-centered visitation. Both patients and family members unanimously expressed support for extending visiting hours. As Participant F5 stated, “I think twice a day would be ideal—once in the morning and once in the afternoon, with each visit lasting 20 to 30 min.” Similarly, Participant P6 shared, “When I’m awake, there is no one to talk to. I look forward to the short afternoon visits every day and hope visiting hours can be extended so I can spend more time with my family.”

Six interviewees commented on the physical environment and layout of the ICU. Participant D1 noted, “Single-room units could be considered for implementing family-centered visitation, as they help ensure patient privacy and may improve the prevention of nosocomial infections.” Participant N4 suggested, “A disinfection area could be set up before entering the room to allow thorough head-to-toe disinfection, enabling family members to have direct contact with the patient.” Participant N16 proposed, “A family rest area could be established near the ward entrance, equipped with health education materials to serve as a channel for health knowledge dissemination.”

All respondents advocated for incorporating social support—from relatives, friends, peers, counseling services, and hospice care—into the family-centered visitation model. Participant P5 stated, “I wish my family could stay with me longer. Their care and support strengthen my confidence in recovery.” Participant D2 emphasized, “Patients need support not only from family members but also from healthcare professionals. Discussing the condition during visits can aid recovery.” Participant N7 added, “Patients and families may experience psychological distress such as anxiety and depression after ICU discharge. Providing psychological support can help mitigate such distress.”

All interviewees acknowledged the importance of palliative care support. Participant N3 observed, “Currently, there is limited emphasis on palliative care in clinical practice. Given the high mortality rate in the ICU, integrating palliative care into ICU settings will undoubtedly be a future direction of development.”

### 3.4 Theme 2: communication and information sharing

The rapid advancement of information technology has introduced “big data” into healthcare, facilitating the development of intelligent, multifunctional, and multi-platform information delivery systems for clinical communication. All participants expressed a desire for access to patient information through multiple channels and diverse methods.

Three interviewees raised the idea of an ICU diary. Participant N9 noted, “An ICU diary could record information not directly observable by families, offering them an alternative way to understand the patient’s condition.” Participant D5 commented, “Maintaining an ICU diary for long-stay patients represents a humanized approach. Documenting the patient’s daily progress through photos and narratives can enhance the patient’s confidence in recovery and allow the family to perceive the hospital’s compassion.”

The designation of a family coordinator was highlighted as a key factor in ensuring effective information sharing. Participant D4 stated, “If families are to be deeply involved, a primary caregiver should be designated. Learning to care for a patient takes time, and frequent changes in family caregivers would hinder communication.” Participant D8 added, “Having a primary caregiver allows for targeted training and guided involvement, which also facilitates the patient’s early rehabilitation.”

Several participants indicated that traditional methods of explaining medical conditions remain the primary channel for conveying patient information to families, and that family meetings continue to be the main format for such communication. Participant D9 noted, “The most ideal and effective communication method remains direct conversation with the physician. Although electronic platforms provide families with test results and other information, they often cannot interpret these findings and may turn to online sources, where inconsistent information can heighten anxiety. Family meetings remain an essential communication mechanism.” Participant N10 added, “After each family visit, numerous questions tend to arise. We consistently recommend that families communicate directly with the attending physician.”

### 3.5 Theme 3: participation

All participants provided insights into family involvement in family-centered care. Seven healthcare professionals noted that family members could appropriately assist with activities of daily living (ADL) care, as opposed to specialized clinical procedures. As Participant N15 explained, “I believe family members could assist with ADL such as shampooing, massaging, and sponge bathing. This can help avoid patient embarrassment while allowing them to feel cared for by their relatives.” Participant D5 remarked, “Early mobilization is currently a key focus in clinical research. Guiding patients to get out of bed can reduce ICU length of stay and promote recovery. Under the guidance of medical staff, encouraging primary caregivers to participate in early mobilization is crucial.” Participant F5 expressed, “Being able to do something for the patient is also a comfort to us as family members.” Participant P9 noted, “I would like my family to be involved in care activities, as this allows more time together and enables them to learn some care skills.” Participant D4 emphasized, “For families to participate effectively, relevant training must first be provided through education and guidance. Only when family members possess certain care competencies can family-centered care be fully implemented.”

Regarding family involvement in rounds, crisis management, and shared decision-making, most participants considered it moderately feasible and important. Participant D2 commented, “We use professional terminology during rounds that families may not understand. Having families present could make us more cautious in our discussions, potentially interfering with the standard rounding protocol.” Participant N4 expressed, “I am uncertain whether every family member has the capacity to assess the patient’s condition. Such differences in perception could potentially exacerbate patient-provider conflicts.” Participant N13 suggested, “If a patient has some medical knowledge, they could be invited to join rounds and participate in shared decision-making.” Participant F1 stated, “Participating in rounds would allow more communication with doctors and provide an additional channel for understanding the patient’s condition.” Participant P8 reflected, “My family lacks medical knowledge. Involving them in rounds and shared decision-making could impose an additional psychological burden on them.”

## 4 Discussion

### 4.1 The imperative for developing a family-centered visiting model: rationale and foundations

With the evolution of the medical paradigm, clinical priorities in the Intensive Care Unit (ICU) have expanded beyond a sole focus on patient survival to encompass long-term health outcomes and quality of life. The family-centered visitation model promotes flexible visiting arrangements while being integrated into a structured clinical management framework. This model requires healthcare professionals to develop individualized visitation plans based on patient condition assessments, incorporating the specific needs of both patients and their families. In this process, visitation safety represents an essential prerequisite for the model’s effective implementation.

Internationally, family-centered visitation systems are well-established and have demonstrated efficacy in promoting family involvement in patient care, optimizing resource allocation, improving clinical decision-making, and enhancing satisfaction among patients and families. However, due to differences in national contexts and healthcare systems, certain international practices are not directly applicable to the Chinese setting. Currently, there is a lack of systematic, localized research in China to support the effective implementation of such a model. Therefore, this study aims to explore an ICU visitation system adapted to China’s healthcare environment and cultural background. It seeks to improve the overall quality of life for critically ill patients and their families and to provide an evidence-based foundation for developing relevant policies and practices.

### 4.2 A comprehensive feasibility assessment of family-centered visitation in the ICU

Research on the family-centered care model in China began relatively late. In recent years, domestic studies have primarily focused on visitation models, discharge planning, and hospice care, with most of this research targeting patients in the Neonatal Intensive Care Unit (NICU) or Pediatric Intensive Care Unit (PICU). Domestic studies have shown that involving family members in patient care—assisting with daily living activities and establishing WeChat groups to provide informational support—can effectively reduce the length of ICU stay and the duration of mechanical ventilation for patients (15, 16). Regarding visitation, some domestic researchers have experimented with extending visitation hours in the NICU and guiding family members to participate in daily care activities such as feeding, cleaning, and bathing, as well as in monitoring patient conditions, including nebulization, temperature monitoring, and bowel movements. These interventions have significantly improved family satisfaction (17, 18). However, there is still no systematic application of family-centered care for adult ICU patients in China, and several barriers remain to implementing family-centered care and visitation in domestic settings.

In interviews, healthcare professionals indicated that implementing a family-centered visitation system requires the provision of single-patient rooms to ensure privacy protection. Establishing such rooms is considered effective in reducing the incidence of hospital-acquired infections while providing a comfortable environment for family members (11). It is recommended that units with adequate environmental facilities attempt to implement family-centered visitation and care. Although extensive clinical studies have confirmed that visitation time in the Intensive Care Unit (ICU) is not correlated with the rate of acquired infections (12), healthcare professionals remain concerned about potential increases in ward staffing, which could significantly raise the risk of hospital-acquired infections. Research indicates that the rise in hospital infection rates is primarily attributed to inadequate hand hygiene and insufficient isolation measures (13). Therefore, key strategies for preventing hospital-acquired infections include ensuring that family members wear isolation gowns and follow standard handwashing procedures before entering the ICU, implementing effective contact isolation, maintaining good air circulation in patient rooms, and ensuring thorough and continuous environmental cleaning and disinfection. These measures provide a foundation for the successful implementation of family-centered care and visitation.

Furthermore, to successfully carry out family-centered care and visitation, policy support is essential. Ensuring an adequate number of healthcare professionals is a prerequisite for facilitating family involvement.

### 4.3 Enhancing the pathways for information sharing

The critical condition of ICU patients, coupled with the limited clinical experience of newly appointed nursing staff, can complicate care delivery. In contrast, primary caregivers are often more adept at communicating the patient’s condition and, over time, become capable of participating in shared decision-making and ward visits. Familiarity between healthcare providers and primary caregivers can enhance mutual trust and foster mutual understanding.

Family-centered visitation constitutes a novel multidisciplinary bedside model, the core of which involves actively engaging ICU patients’ families (9). The inclusion of family members in medical visits facilitates surrogate decision-making and strengthens their involvement in patient care (19). Some studies suggest that a lack of medical knowledge or insufficient family engagement may prolong hospital stays and increase the risk of medical disputes, thereby hindering the implementation of the family-centered visitation model (20). However, international nursing research indicates that family participation does not reduce the efficiency of clinician–patient interactions; rather, it can shorten the duration of family meetings and enhance the effectiveness of medical discussions (21).

Shared decision-making in the ICU is a collaborative process that involves healthcare professionals, patients, and their families in reaching consensus on treatment plans based on the principles of family-centered care (22). This approach prioritizes the needs and values of patients and their families, supports evidence-based evaluation of treatment benefits and risks, and ensures through structured dialogue that the patient’s best interests are fully considered (23). Research shows that shared decision-making between families and clinicians can alleviate the decision-making burden on families and improve the quality of decisions (24).

Although clinical guidelines advocate for shared decision-making, empirical evidence in the domestic context suggests that only a small proportion of physicians actively integrate patient values and preferences into clinical practice (25). In reality, clinical decisions remain largely clinician-led. Therefore, it is recommended that domestic physicians recognize the critical role of primary caregivers, guide their participation in appropriate clinical activities, provide structured support to families, and promote positive health outcomes for both providers and patients.

### 4.4 Foster family participation in care delivery

The family-centered visitation model promotes family involvement in patient care and early rehabilitation. Research indicates that in the high-stress ICU environment, family members are frequently exposed to significant pressure, which may impair their capacity to provide effective care (26). Multiple studies have confirmed the feasibility and effectiveness of the involvement of families in patient care (27). Moreover, ICU healthcare teams implement supportive interventions and targeted training programs to enhance family members’ caregiving skills, and thus promote their active engagement in the care process. Such involvement not only empowers families but also enhances their comprehension of communication and family-centered healthcare services (28).

This study found that most patients expressed a desire for family companionship and care within the ward, as this helps alleviate psychological distress. Additionally, most medical staff supported family involvement in patients’ daily care, emphasizing the need for proactive health guidance provided in diverse formats. This approach demonstrates strong feasibility and significant potential for development in the domestic context. However, regarding family presence during resuscitation, both international research and this study indicate predominantly cautious or negative attitudes among healthcare professionals and families, reflecting persistent disagreements and barriers to family involvement in critical care.

In China, the family-centered visitation system remains exploratory, with existing research primarily focused on neonatal and pediatric wards. This study aims to investigate the applicability of the family-centered visitation model in adult ICUs and to elucidate the prevailing perceptions of this approach among ICU medical staff, patients, and their families.

## 5 Conclusion

This qualitative study has elucidated the multifaceted perceptions of key stakeholders regarding the implementation of a family-centered visitation model within the Chinese ICU setting. It reveals a shared acknowledgement of its potential benefits as well as significant concerns regarding workforce capacity, environmental constraints, and clinical management. To bridge the gap between principle and practice, future efforts should transition from broad advocacy to concrete action. We recommend that hospital administrators initiate tailored pilot programs that include structured staff training and the development of specific infection-control and family-participation guidelines, thereby cultivating an environment conducive to sustainable implementation. Subsequent research should empirically evaluate the impact of these targeted interventions to establish a robust evidence base for policy development, with the ultimate aim of fostering a more humanistic and collaborative paradigm of care in ICUs across China.

### 5.1 Limitations and strengths

The external validity of the findings from this study is limited by its single-center design. Future multi-center research is warranted that would enhance the robustness and generalizability of the conclusions.

Additionally, the limited sample size in this investigation precluded a thorough examination of the relationships between demographic characteristics and study outcomes. Subsequent studies should therefore utilize larger and more diverse cohorts to validate these preliminary findings.

Future research must prioritize clinical trials adapted to China’s specific healthcare context and institutional realities to evaluate the implementation outcomes and address the practical challenges of the family-centered visitation system. Such research is essential for establishing a robust evidence base to inform national policies and clinical guidelines, and thereby facilitate the effective dissemination of this model across ICUs in China.

## Data Availability

The original contributions presented in the study are included in the article/supplementary material, further inquiries can be directed to the corresponding authors.

## Appendix: Interview protocol

Introduction: The objective of this research is to comprehend the attitudes of diverse stakeholder groups within the Intensive Care Unit (ICU) toward family-centered visitation policies and care paradigms. The interview is anticipated to require 30 to 45 min of your time. To ensure the fidelity and comprehensiveness of data collection, the session will be audio-recorded in its entirety. We hereby assure you of the strictest confidentiality regarding the information you disclose. All data will be utilized exclusively for the purposes of this academic investigation. Your identity will be anonymized through the use of a numerical code, and no personally identifiable information, including your name, will appear in any research outputs. Subsequent to the conclusion of the study, all audio recordings will be systematically and permanently destroyed. Please be advised that your participation is voluntary; you retain the right to decline to answer any question or to withdraw from the interview at any point without incurring any prejudice or adverse consequences.

(1)Interview Protocol for Healthcare Professionals:

(1) What are your perspectives regarding the current visitation protocols implemented within the ICU?
(2) What modifications or improvements would you propose for the existing ICU visitation system?
(3) Are you acquainted with the conceptual framework of family-centered visitation?
(4) In your professional opinion, what constitute the principal barriers or impediments to the implementation of such a family-centered visitation model?
(5) What methodologies or channels do you deem effective for facilitating and enhancing communication with patients and their family members?
(6) Which specific nursing or care activities do you believe family members could appropriately participate in during visitation periods?

(2) Interview Protocol for Patients:

(1) Could you elaborate on your subjective experiences and overall perceptions during your stay in the ICU?
(2) What are your suggestions or viewpoints concerning the daily visitation arrangements you encountered?
(3) What specific actions or forms of support would you have desired from your family members during their visits?
(4) What aspects of the ICU visitation experience do you believe warrant improvement in the future?

(3) Interview Protocol for Family Members:

(1) What was your personal, emotional experience while your relative was hospitalized in the ICU?
(2) What specific information did you feel the need to acquire during that period?
(3) Through which preferred channels or modes of communication would you have wished to receive such information?
(4) How do you evaluate the current visitation regulations?
(5) What activities or care tasks did you aspire to perform for your relative during the allotted visitation times?
(6) In your view, what enhancements or reforms are necessary regarding future visitation practices in the ICU?
